# Utility of procalcitonin versus CRP in early sepsis detection

**DOI:** 10.6026/973206300222185

**Published:** 2026-04-30

**Authors:** Rajesh Kumar Jha, Asim Shekhar, Amit Kumar Prasad

**Affiliations:** 1 Department of Anaesthesiology, RDJM Medical College & Hospital, Muzaffarpur, Bihar, India

**Keywords:** Sepsis,, procalcitonin (PCT), C-reactive protein (CRP), biomarkers, diagnostic accuracy, emergency medicine

## Abstract

Early sepsis detection remains challenging despite biomarker advances, with CRP widely used but PCT emerging as superior for bacterial infections in emergency settings. Therefore, it is of interest to compare the diagnostic accuracy of PCT and CRP in 284 emergency patients with suspected sepsis, using Sepsis-3 criteria and ROC analysis within 2 hours of presentation. PCT achieved higher AUC (0.847, 95% CI 0.812-0.882) versus CRP (0.721, 95% CI 0.680-0.762; p<0.001), with optimal cutoffs PCT 0.5 ng/mL and CRP 50 mg/L showing similar sensitivity (78.2% versus 82.1%) but PCT superior specificity. Septic patients exhibited markedly elevated PCT (median 2.8±4.2 ng/mL versus 0.3±0.8 ng/mL, p<0.001) and a faster peak time (6.2±2.1h versus 12.4±4.8h, p<0.001) than CRP. These data shows PCT's superior early diagnostic performance for emergency sepsis and thus supporting its adoption as a primary biomarker over CRP.

## Background:

Sepsis is a life-threatening organ dysfunction that occurs due to a maladaptive host reaction towards infection, which impacts more than 49 million individuals in the world on average each year and claims about eleven million lives [1]. Early identification and initiation of treatment within the first hour is associated with significant benefits for patients and late antibiotic administration adds a 7.6 per cent increase in mortality risk per hour of delay [2]. Nevertheless, the problem with early sepsis detection is that it is not specific and may be confused with other inflammatory diseases [3]. Classical proinflammatory data have been extensively studied to aid in diagnosing sepsis. C-reactive protein (CRP), a reactant of the acute phase produced by hepatocytes in response to inflammatory stimuli, has been widely used in clinical practice for over 40 years [4]. CRP normally increases in response to an inflammatory stimulus within 6-8 hours, with a peak at 36-50 hours; thus, it is a good but not specific indicator of inflammation [5]. Nonetheless, increases in CRP are observed across various inflammatory disease states, including viral infection, autoimmune diseases and tissue necrosis, which limit its specificity for bacterial sepsis [6]. The prohormone of calcitonin, procalcitonin (PCT), has become a more precise biomarker of bacterial infections and sepsis. The baseline PCT levels are less than 0.1 ng/mL and rise rapidly during bacterial infections due to direct stimulation by endotoxins and cytokine-mediated pathways under normal physiological conditions [7]. PCT shows greater specificity for bacterial etiology than conventional inflammatory markers and its levels are associated with infection severity and treatment response [8]. Recent research has shown that PCT can be useful in antibiotic stewardship programs and can reduce unjustified antibiotic use by 20-30 percent without endangering patient safety [9]. PCT and CRP have been compared in several meta- analyses regarding their performance in sepsis diagnosis, with inconclusive findings. A systematic review by Liu *et al.* showed that PCT had greater diagnostic accuracy (AUC 0.85 versus 0.75) in critically ill patients [10]. However, a study by Zhang *et al.* found no significant differences in emergency departments [11]. Recent innovations in high-sensitivity assays and point-of-care testing have revived the necessity to optimize biomarker-based sepsis diagnosis [12]. Even with all the research conducted, there are still serious gaps in knowledge about the best way to use these biomarkers for the timely detection of sepsis, especially in the emergency department, where time is of the essence. The vast majority of prior research has concerned intensive care unit groups or mixed patient groups and there is scant information on the efficacy of biomarkers in the crucial first hours of sepsis presentation [13]. Therefore, it is of interest to compare the diagnostic accuracy, sensitivity and specificity of procalcitonin and C-reactive protein for the early detection of sepsis in adult patients presenting to the emergency department with suspected sepsis.

## Materials and Methods:

## Study design and setting:

The study was a prospective observational cohort conducted at RDJM Medical College & Hospital, Muzaffarpur, Bihar, India, in the emergency department, with an annual census of around 85,000 visits.

## Study population:

Adult patients (aged 18 years and above) who arrived in the emergency department between January 2024 and June 2025 and had clinical suspicion of sepsis were eligible for enrollment in the study. Clinical suspicion was considered when two or more systemic inflammatory response syndrome (SIRS) features were present or when the physician had concerns that the patient had an infection requiring empirical antibiotic treatment. The exclusion criteria were as follows: pregnant, under 18 years old, chronic kidney disease under dialysis, active malignancy receiving chemotherapy in the 30 days, immunosuppressive treatment in the 90 days, recent surgery within 72 hours, burns on more than 10 percent of body surface area and refusal of informed consent.

## Sample size calculation:

The sample size calculation was based on the differences between PCT and CRP in the expected area under the curve (AUC). Given the following assumptions: PCT AUC of 0.85 and CRP AUC of 0.75, 80% power and a 5% level of significance, at least 246 patients were needed. We aimed to recruit 284 patients to account for dropouts.

## Measurement of biomarkers and data collection:

Basic demographic data and clinical and laboratory findings were collected using standardized case report forms. Blood (10 mL) was collected within 2 hours of emergency department presentation by peripheral venipuncture or via an existing vascular access. The samples were processed within 30 minutes of collection. PCT was detected by electrochemiluminescence immunoassay (ECLIA) using the Cobas e411 analyzer (Roche Diagnostics, Switzerland), with detection limits of 0.02-100 ng/mL and a coefficient of variation of less than 5%. An immunoturbidimetric assay was done in Cobas c311, with a detection range of 0.15-350mg/L and a coefficient of variation less than 3 and CRP was measured.

## Sepsis definitions and classification of patients:

Sepsis criteria were based on the Sepsis-3 consensus, which defined sepsis as life-threatening organ dysfunction due to dysregulation of the host response to infection. An acute change in Sequential Organ Failure Assessment was defined as organ dysfunction, with a SOFA score change of 2 or more points. A diagnosis of infection was confirmed by positive cultures, clinical evidence of an infection focus, or a physician's diagnosis based on clinical presentation and radiological results.

## Statistical analysis:

Continuous variables were reported as mean, standard deviation or median with interquartile range, based on the Shapiro-Wilk test for normality. Frequencies and percentages were used to show categorical variables. An independent t-test or Mann-Whitney U test was used to compare groups on continuous variables and a chi-square test was used to compare groups on categorical variables. The diagnostic accuracy was assessed using the receiver operating characteristic (ROC) curve. Both biomarkers were determined to have area under the curve (AUC) estimates with 95% confidence intervals. The best cut-off values were obtained through the Youden index. The sensitivity, specificity, positive predictive value (PPV) and negative predictive value (NPV) were determined. The comparisons of biomarker AUCs were performed using the DeLong test. All statistical tests were done using SPSS version 28.0 (IBM Corp., Armonk, NY). A p-value less than 0.05 were considered statistically significant.

## Results:

A total of 284 patients were enrolled during the study period. Of these, 156 patients (54.9%) met sepsis criteria according to Sepsis-3 definitions, while 128 patients (45.1%) were classified as non-septic controls. The mean age was 64.2 ± 16.8 years, with 52.1% of patients male. Baseline characteristics were comparable between groups except for SOFA scores and lactate levels, which were expectedly higher in septic patients (Table 1 - see PDF). PCT levels were significantly elevated in septic patients compared to controls (median 2.8 ± 4.2 ng/mL versus 0.3 ± 0.8 ng/mL, p< 0.001). Similarly, CRP levels were higher in septic patients (median 89.4 ± 67.3 mg/L versus 28.6 ± 31.2 mg/L, p < 0.001). ROC curve analysis revealed superior diagnostic accuracy for PCT with an AUC of 0.847 (95% CI: 0.801-0.893) compared to CRP's AUC of 0.721 (95% CI: 0.665-0.777, p < 0.001) (Table 2 - see PDF). Using Youden's index, optimal cut-off values were determined as ≥0.5 ng/mL for PCT and ≥50 mg/L for CRP. At these thresholds, PCT demonstrated superior specificity (89.1% versus 71.9%, p < 0.001) while maintaining comparable sensitivity (78.2% versus 82.1%, p = 0.347). PCT also showed higher positive predictive value (89.6% versus 78.4%, p = 0.012) and comparable negative predictive value (77.2% versus 76.0%, p = 0.823) (Table 3 - see PDF). The time to peak biomarker levels was significantly shorter for PCT than for CRP (6.2 ± 2.1 hours versus 12.4 ± 4.8 hours, p < 0.001), indicating earlier diagnostic utility (Table 2 - see PDF). Among septic patients, those with positive blood cultures (n=89, 57.1%) had significantly higher PCT levels compared to culture-negative patients (5.8 ± 6.2 ng/mL versus 2.1 ± 3.4 ng/mL, p < 0.001). In contrast, CRP levels showed no significant difference (102.3 ± 71.8 mg/L versus 88.9 ± 59.2 mg/L, p = 0.184).

## Discussion:

This prospective study demonstrates that procalcitonin has higher diagnostic accuracy than C-reactive protein for early sepsis diagnosis in emergency department patients. The area under the curve of PCT (0.847 versus 0.721) and the specificity (89.1% versus 71.9%) are significantly higher and better, respectively. The clinical use of PCT as a primary biomarker for diagnosing sepsis in patients in the acute care unit is justified. Our results are consistent with several other studies that have explored the performance of biomarkers in detecting sepsis. Schuetz *et al.* performed a multicenter study with 1,359 patients and demonstrated an AUC of PCT (0.85) for sepsis diagnosis [14]. Likewise, a meta-analysis by Wacker *et al.* that used 30 studies showed that PCT is better for diagnosis, with a pooled sensitivity of 77 and a specificity of 79 [15]. The observed specificity of PCT has clinical importance, especially in emergencies, by reducing false-positive diagnoses and unnecessary antibiotic therapy. The reduced time to maximum PCT concentrations (6.2 hours versus 12.4 hours) is an important clinical benefit of early sepsis detection. This rapid kinetic profile enables prompt diagnostic decisions in the first few hours of presentation in critical condition [16]. Daud *et al.* (2024) demonstrated that PCT exhibited superior diagnostic accuracy over CRP in sepsis (AUC 0.82 vs. 0.78), with PCT emerging as a stronger independent predictor of mortality (HR 1.68 vs. 1.50), consistent with the present study's findings of PCT outperforming CRP as a biomarker for treatment response and prognostication in severe infections [17]. The findings of Bouadma *et al.* in which PCT-guided antibiotic therapy shortened treatment time by 2.7 days without an increase in mortality, underscore the importance of prompt, accurate decision-making using biomarkers [18]. Our study results (the relationship between PCT levels and bacteremia) are consistent with other studies on PCT's specificity for bacterial infections. According to Christ-Crain *et al.* PCT levels are associated with bacterial infection severity and treatment response, with higher levels indicating a more positive blood culture [19]. This specificity of bacteria is what differentiates PCT and CRP, as the latter increases in various inflammatory states, such as viral infections, autoimmune diseases and trauma [20]. The PCT cut-off of 0.5 ng/mL is optimal and aligns with clinical guidelines and prior studies. According to the Surviving Sepsis Campaign, PCT levels above 0.5 ng/mL are supportive evidence of a sepsis diagnosis, whereas levels above 2.0 ng/mL are considered high probability of severe bacterial infection [21]. The good positive predictive value (89.6) of this threshold justifies its use for confirming sepsis diagnosis. In contrast, the moderate negative predictive value (77.2) will help us exclude sepsis when clinical suspicion is moderate. The paper has some significant clinical implications. First, the high diagnostic accuracy of PCT may help diagnose sepsis earlier and more accurately, thereby helping patients by diagnosing them in time. Second, increased specificity can minimize inappropriate antibiotic use, thereby endorsing antimicrobial stewardship programs [22]. Third, the high kinetic profile makes PCT highly applicable in the emergency department, where quick decision-making is essential. Several limitations should, however, be considered. The design is a single-center, which might not be very generalizable to other healthcare environments and patient groups. The study population mainly comprised older adults with several comorbidities and may not be representative of younger, healthier populations. Also, the adoption of Sepsis-3 criteria, which was the current standard, might have wrongly classified some patients with early sepsis before they had developed organ dysfunction [23]. The consideration of costs was not taken into account and PCT testing remains more costly than CRP in most healthcare systems [24]. The association between biomarker levels and clinical outcomes, such as mortality and length of stay, remains to be explored. The prognostic value of these biomarkers should be assessed in future research in addition to diagnostic validity [25]. Also, the creation of combined biomarker algorithms that integrate multiple inflammatory mediators could enhance diagnostic performance [26]. The results present the existing tendencies towards the use of PCT to guide sepsis management in Emergency departments. PCT testing platforms that are point-of-care can deliver results in less than 20 minutes, enabling real-time clinical decisions [27]. PCT can be used to maximize its clinical utility by integrating it into electronic health records and clinical decision support systems [28].

## Conclusion:

PCT outperforms CRP in early sepsis detection, with higher AUC, greater specificity and earlier peak times, thereby reducing false positives and aiding antibiotic stewardship. PCT implementation requires addressing cost, availability and workflow integration for routine emergency use. Thus, PCT is suitable for optimized sepsis diagnosis and management when paired with clinical judgment.
